# Intra- and Inter-Rater Reliability of Manual Feature Extraction Methods in Movement Related Cortical Potential Analysis

**DOI:** 10.3390/s20082427

**Published:** 2020-04-24

**Authors:** Gemma Alder, Nada Signal, Usman Rashid, Sharon Olsen, Imran Khan Niazi, Denise Taylor

**Affiliations:** 1 Health and Rehabilitation Research Institute, Auckland University of Technology, Auckland 0627, New Zealand; nada.signal@aut.ac.nz (N.S.); usman.rashid@aut.ac.nz (U.R.); sharon.olsen@aut.ac.nz (S.O.); imran.niazi@nzchiro.co.nz (I.K.N.); denise.taylor@aut.ac.nz (D.T.); 2 Centre for Chiropractic Research, New Zealand College of Chiropractic, Auckland 1060, New Zealand

**Keywords:** electroencephalography (EEG) processing, event related potential (ERP), movement related cortical potential (MRCP), stroke, intra-rater reliability, inter-rater reliability

## Abstract

Event related potentials (ERPs) provide insight into the neural activity generated in response to motor, sensory and cognitive processes. Despite the increasing use of ERP data in clinical research little is known about the reliability of human manual ERP labelling methods. Intra-rater and inter-rater reliability were evaluated in five electroencephalography (EEG) experts who labelled the peak negativity of averaged movement related cortical potentials (MRCPs) derived from thirty datasets. Each dataset contained 50 MRCP epochs from healthy people performing cued voluntary or imagined movement, or people with stroke performing cued voluntary movement. Reliability was assessed using the intraclass correlation coefficient and standard error of measurement. Excellent intra- and inter-rater reliability was demonstrated in the voluntary movement conditions in healthy people and people with stroke. In comparison reliability in the imagined condition was low to moderate. Post-hoc secondary epoch analysis revealed that the morphology of the signal contributed to the consistency of epoch inclusion; potentially explaining the differences in reliability seen across conditions. Findings from this study may inform future research focused on developing automated labelling methods for ERP feature extraction and call to the wider community of researchers interested in utilizing ERPs as a measure of neurophysiological change or in the delivery of EEG-driven interventions.

## 1. Introduction

Surface electroencephalography (EEG) has been widely used to study electrophysiological activity within the cortex [1]. A common approach is to record event-related potentials (ERPs), which are often recorded following the presentation of a sensory stimulus, and are characterized by a range of waveforms of varying latencies and amplitudes which are time-locked to specific sensory, motor, or cognitive events [2]. ERPs have considerable epoch-to-epoch variability, due to random noise and artefacts [3]. To account for this, ERPs are often processed by averaging a number of epochs (between 50 and 200) [3,4,5,6]. To ensure each single epoch contains an ERP, and not an artefact, each epoch is visually inspected prior to averaging, then the average ERP is visually inspected, and relevant features of the waveform manually labelled [5,6,7,8,9]. This process of visually inspecting and manually labelling ERP data is common practice [5,6,7,8,9] but little is known about its reliability.

In the field of diagnostics, the inter-rater reliability of visual-inspection methods for identifying abnormal activity in continuous EEG data have shown only fair to moderate reliability [10,11,12]. The few studies which have investigated the inter-rater reliability of visual inspection and manual labelling have demonstrated moderate to excellent inter-rater reliability using a range of statistical methods across a variety of ERP waveforms [13,14]. No studies were identified which evaluated intra-rater reliability. Those studies comparing the reliability of human experts with that of algorithms suggest that better agreement is seen between humans, than between algorithms, or between humans and algorithms [13,14]. Given the growing use of EEG data within research settings, for both outcome measurements [13] and the delivery of EEG-driven interventions [4,15,16], it is important to assess the reliability of manual ERP labelling methods, prior to establishing effective algorithms that can automatically carry out this process. 

An example of a context which requires reliable labelling of ERPs is in the delivery of an endogenous paired associative stimulation (ePAS) intervention, a non-invasive neuromodulatory intervention that has been shown to modulate corticomotor excitability in healthy people [7,17,18,19] and people with stroke [20,21]. Based on traditional paired associative stimulation (PAS) [22,23,24], ePAS involves the delivery of 50 single pulses of peripheral electrical stimulation, each paired with an endogenous ERP signal known as the movement related cortical potential (MRCP) [7,17,18,20,21]. The MRCP is observed as an individual prepares and executes a voluntary or imagined movement [25,26] and is characterized by a slow (≈0.5 Hz) negative potential which begins approximately 1.5 to 2 s prior to movement and peaks around the onset of movement (amplitude –5 to –30 uV) [25,26]. When the MRCP is generated during externally-cued movements (i.e., where there is a “Warning” and a “Go” cue), it is also known as a contingent negative variation [27].

The neuroplastic effects of ePAS rely on accurately timing the peripheral electrical stimulation so that the afferent volley arrives in the primary motor cortex (M1) at the most negative point of the MRCP (peak negativity (PN)) [7]. This timing is achieved by triggering the peripheral electrical stimulation at a set number of milliseconds before the PN of the MRCP to account for the conduction time between the peripheral nerve and the M1 [7]. However, the neuromodulatory effects of ePAS present considerable between-participant variability [17,18,19,20,21]. For example, Olsen et al. [17] showed increases in corticomotor excitability ranging from 4%–396% (*n* = 10). While large between-participant variability has been documented in response to other neuromodulatory interventions [28,29,30,31,32,33,34,35] it is important to identify sources of variability that can be controlled [29] such as inconsistencies in the intervention delivery method [29,31,34]. One factor that may contribute to the variable response following ePAS, is inaccurate estimation of the PN of the averaged MRCP [7]. This currently involves visual inspection of 50 pre-recorded epochs, exclusion of epochs that lack the expected MRCP characteristics, and then labelling of the PN from the averaged MRCP. Any variability in this process will alter the PN value, which is used to time the peripheral electrical stimulation in the subsequent ePAS intervention potentially influencing efficacy [7]. The primary aim of this study was to examine the intra-rater and inter-rater reliability of EEG experts’ identification of the PN timing from averaged MRCPs derived from healthy people and people with stroke.

## 2. Method

### 2.1. Study Design

**Figure 1:**
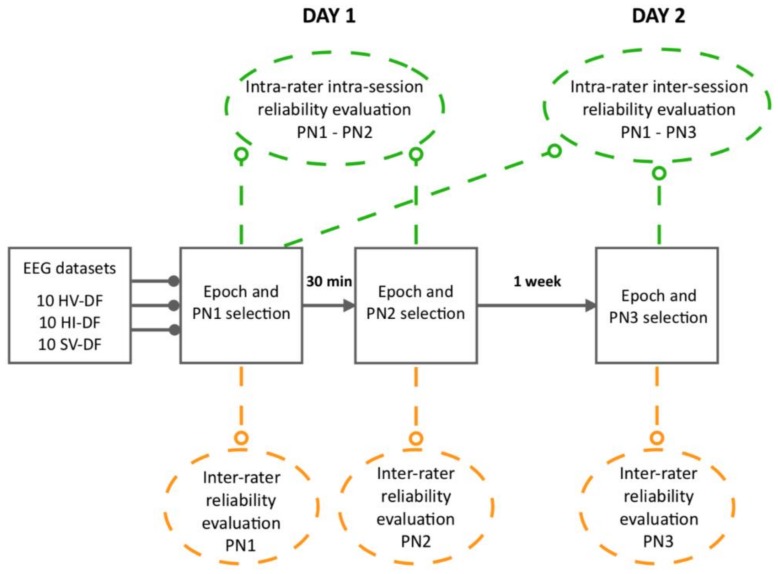
An overview of the study design. The three conditions and the 10 datasets within a condition were pseudo-randomized for each expert at each separate evaluation session. Epochs within a single dataset remained in the order in which they were recorded. HV-DF = healthy voluntary dorsiflexion; HI-DF = healthy imagined dorsiflexion; SV-DF = stroke voluntary dorsiflexion. PN1 = evaluation session 1 day 1; PN2 = evaluation session 2 day 1; PN3 = evaluation session 3 day 2.

This reliability study utilised a repeated-measures design (refer to Figure 1). Five EEG experts participated in three separate MRCP evaluation sessions: sessions 1 and 2 were conducted on the first day with a 30-min break in-between, and session 3 was conducted seven days later. Intra-rater reliability for each expert was assessed within (intra-session) and across evaluation sessions (inter-session). Inter-rater reliability across experts was assessed within each of the three evaluation sessions. At each session, experts examined the same 30 EEG datasets including three different conditions: (1) healthy participants performing 50 voluntary ankle dorsiflexion movements (10 datasets); (2) healthy participants performing 50 imagined ankle dorsiflexion movements (10 datasets); and (3) participants with stroke performing 50 voluntary ankle dorsiflexion movements (10 datasets). Each dataset contained 50 epochs, relating to 50 repetitions of movement. The experts visually examined each epoch using a custom-built graphical user interface tool developed in MATLAB 2016a and manually excluded epochs they considered not representative of the expected MRCP characteristics. The included epochs within each dataset were then averaged to produce an average MRCP and experts were required to manually label the peak negativity (PN).

### 2.2. Participants

EEG experts were required to have had at least one years’ experience working with MRCP signals and the ePAS intervention protocol. EEG data were collected from 20 healthy participants and five participants with stroke as part of two independent ePAS studies. The criteria for inclusion of healthy participants were related to age (>18 years) and the absence of neurological conditions. Participants with stroke were included if they had experienced a single stroke at least six months previously, had a hemiparesis impacting their walking ability, and a gait speed of 0.5–1.2 m per second. Participants with stroke were excluded if they had contra-indications to transcranial magnetic stimulation (this related to the ePAS outcome measures), or cognitive, perceptual or communications impairments. Study participant characteristics are reported in Table 1. Healthy participants completed a single MRCP recording session of either visually-cued voluntary dorsiflexion movements (*n* = 10) or visually-cued imagined dorsiflexion movements (*n* = 10). Participants with stroke (*n* = 5) participated in two identical MRCP recording sessions at least three days apart and performed visually-cued voluntary dorsiflexion movements (10 datasets).

### 2.3. Ethical Procedures

EEG experts came from two institutions, the Health and Rehabilitation Research Institute at Auckland University of Technology, Auckland, New Zealand, and the Centre of Sensory-Motor Interaction, Department of Health Science and Technology Alborg University, Denmark. Ethical approval for the two studies from which the data was drawn received ethical approval from the Auckland University of Technology Ethics Committee (AUTEC 15/270 and 14/255). All participants provided written and informed consent prior to participation.

### 2.4. Experimental Procedures

#### 2.4.1. Externally-Cued Movement Paradigm

**Figure 2:**
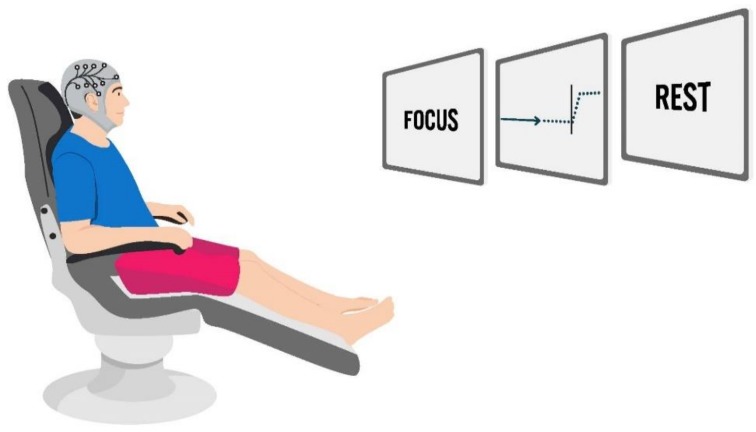
An illustration of the set up for continuous electroencephalography (EEG) recordings where a participant executes either voluntary or imagined ballistic dorsiflexion movements in time with a visual cue displayed on a computer monitor.

Healthy participants and participants with stroke were seated in a chair with approximately 90–100° hip flexion, 25° knee flexion and the ankles in a relaxed slightly plantarflexed position. Participants were orientated to a visual cue on the computer monitor (MATLAB, MathWorks, Inc., Natick, MA, USA, 2011). The visual cue consisted of four different phases that guided participants to (1) focus on the screen, (2) prepare for the movement, (3) execute the ballistic (voluntary or imagined) movement to achieve a fully-dorsiflexed position and hold it for approximately 1 s and then (4) rest. A moving cursor guided the participant through the preparation and execution of movement. The length of focus and rest times varied per repetition (2–3 s and 6–8 s, respectively). Continuous EEG recordings were collected during two sets of 25 repetitions of ankle dorsiflexion movement (voluntary or imagined). Participants with stroke performed the voluntary dorsiflexion to the best of their movement ability, and within their maximal available active dorsiflexion range of motion, which was limited for some participants. An illustration of the laboratory set up is displayed in Figure 2.

#### 2.4.2. EEG Data Acquisition 

A 40 channel EEG Quik-Cap (Compumedics Neuroscan, Dresden, Germany) was fastened below the chin of participants with the Cz electrode positioned midway between the nasion and the inion (sagittal plane), and midway between each tragus (coronal plane). Skin was lightly abraded using a sterile blunt needle. Conductive gel was inserted into 12 electrodes, FP1, F3, Fz, F4, C3, Cz, C4, P3, Pz, and P4, according to the international 10–20 system, as well as a reference electrode on the right mastoid and a ground electrode on the right forehead (Ag/AgCl electrodes, Compumedics, Neuroscan). Impedance remained below 5 kΩ. EEG signals were digitised via a 40-channel EEG amplifier (NuAmps, Compumedics Neuroscan, Dresden, Germany) with a sampling rate of 500 Hz, 32 bits accuracy and recorded in SCAN software (Compumedics Neuroscan, Dresden, Germany).

#### 2.4.3. EEG Data Processing

Continuous EEG signals for each movement condition were imported into MATLAB 2015a. Each channel was band-pass filtered from 0.05 to 5 Hz (second-order zero-phase Butterworth filter). All channels except FP1 were spatially-filtered with a large Laplacian filter with Cz as the centre electrode, to obtain a single virtual channel. The virtual channel was separated into 50, 4.5 s epochs (3 s preceding the cue to move and 1.5 s post) [7,17,36,37].

#### 2.4.4. MRCP evaluation by EEG Experts

Experts evaluated all 30 datasets independently of one another using the customised user interface (MATLAB, MathWorks, Inc., Natick, MA, USA, 2011). Randomization of datasets was performed in MATLAB 2016a using the ***randperm*** function with randomization configuration set to ***shuffle***. This meant that the current time was used as a seed for the random number generator. Conditions, and the 10 datasets within a condition, were randomized for each expert and each separate evaluation session. Epochs within a single dataset remained in the order in which they were recorded from the study participant to reflect normal collection procedures. For each dataset, experts were provided with the following information: the movement type (voluntary or imagined dorsiflexion); the participant population (healthy people or people with stroke); the onset of the ‘cue to move’ within the epochs (at the 1500 samples mark); and the methods by which the EEG data had been recorded and filtered (see Section 2.4.3). Experts were required to visually inspect each of the 50 epochs presented in each dataset and accept or reject epochs based on whether they determined the signal represented an MRCP in view of the information provided. The included epochs within a single dataset were then averaged and presented to the expert as an average MRCP signal. Experts were then required to manually label the PN from the average MRCP signal using the mouse pointer. Experts were instructed to evaluate each dataset as quickly and as accurately as possible.

### 2.5. Statistical Analysis

#### 2.5.1. Primary Analysis: Average MRCP PN Labelling

PN values for each condition within each of the three evaluation sessions were entered into SPSS software package version 24 for analysis. PN data were deemed to be normally distributed based on histogram visual analysis and the Shapiro–Wilks test. Relative reliability for average PN values for each condition was assessed using intraclass correlation coefficient (ICC) estimates. The ICC is a unitless measure that compares the between-participant variance to the total variance [38] which includes between-participant variance and error variance. In this case the error variance represents expert variance, either the variance between experts (inter-rater reliability) or within one expert across two sessions (intra-rater reliability). ICC estimates and their 95% confidence intervals were calculated based on a single measures, absolute agreement, 2-way random-effects model which accounts for both systematic and random error [38,39]. ICC values are bound from 0 to 1, where values closer to 1 indicate stronger reliability. The following criteria were used to interpret ICC values and their 95% confidence intervals: >0.8 excellent; 0.6–0.8 good; 0.4–0.6 moderate; <0.4 poor, [40]. Absolute reliability was assessed using the standard error of the measurement (SEM), which provides an index of reliability in the same unit as the measurement of interest [41], which in the case of PN values, is milliseconds. Unlike the ICC, the efficiency of the SEM is not susceptible to small between-participant variations and quantifies how much the absolute values differ from the ‘true’ value [38]. The smaller the SEM, the greater the reliability. The following equation was used: SEM = √MSE, where MSE represents the mean sum of squares for the error term from the ANOVA [38]. SEM means and standard deviations are reported as mean ± SD for intra- and inter-rater reliability for each condition.

#### 2.5.2. Secondary Analysis: Epoch Selection

We conducted a post hoc secondary analysis to explore factors that may influence the manual selection of epochs by experts. We investigated the influence of two factors on the ability of experts to provide the same response for inclusion of epochs: (1) the morphology of the signal and (2) the experience of the EEG expert. Epoch selection was defined as *‘matched’* if an expert chose to *accept* an epoch for inclusion at two different evaluation sessions (intra-rater: evaluation sessions 1 and 2 and 1 and 3) or if all five experts *accepted* the same epoch in a single evaluation session (inter-rater: evaluation sessions 1, 2 and 3). The morphology of the epochs was quantified using the cosine similarity index. This was defined as the similarity of a single epoch from a participant compared to the average of all 50 epochs from the same participant, which was considered a representation of the expected MRCP characteristics [42],

$$\mathrm{Cosine}\;\mathrm{similarity}\;\mathrm{index}=\frac{u.v}{||u||\;x||v||\;}$$

where u and v are vectors, u is the average MRCP of all 50 epochs from a participant and v is a single epoch from the same participant. ‘.’ represents the dot product between the two vectors and ‖.‖ represents the L2 norm of a vector. The self-reported experience of experts working with MRCP signals was quantified in years. A linear mixed-effects model was set up to evaluate the variance of cosine similarity across conditions. In the case of significant findings for cosine similarity across conditions, pairwise *t*-tests using Tukey’s method were performed and presented with cosine similarity means and standard deviations as mean ± SD for each condition. Logistic mixed-effects models were set up to fit linear trends between the log odds for getting a matched epoch, cosine similarity and expert experience. The log odds were transformed to the corresponding linear scale and presented as the probability of getting a matched epoch. For each condition effect sizes were reported along with their 95% confidence intervals. All models were set up in R version 3.5.1 (R Foundation for Statistical Computing, Vienna, Austria) and fitted using lme4 package version 1.1-17 [43]. For each condition the significance level was set to *p* < 0.05. The statistical models set up to test these secondary hypotheses can be found in Supplementary Materials.

## 3. Results

**Figure 3:**
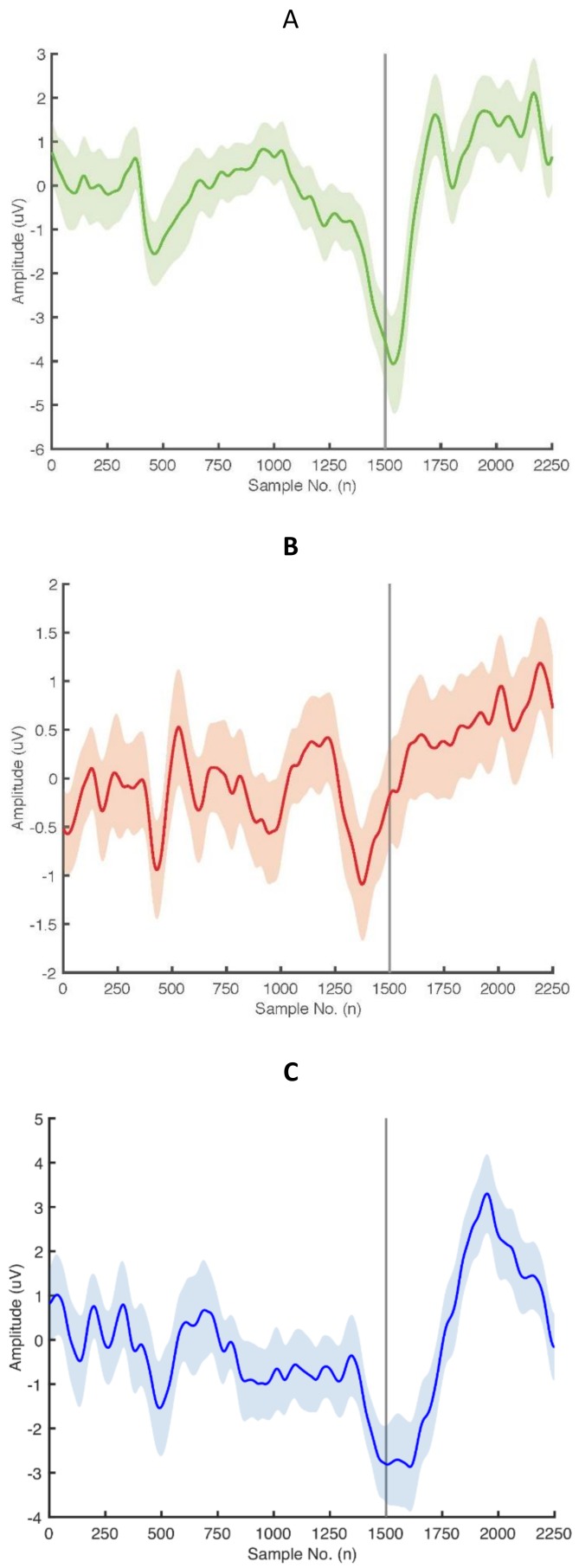
Movement related cortical potential (MRCP) averages with 95% confidence intervals obtained from averaging filtered epochs from (**a**) a healthy participant performing voluntary dorsiflexion, (**b**) a healthy participant performing imagined dorsiflexion and (**c**) a participant with stroke performing voluntary dorsiflexion. Sample number 1500 corresponds to the onset of the cue to move.

The five EEG experts had a mean of 4.7 years of experience (range 1.5–8 years) working with MRCP signals and the ePAS intervention. Figure 3 presents examples of MRCP averages with 95% confidence intervals representing the filtered epochs of one participant from each condition.

### 3.1. Primary Findings: Average MRCP PN Labelling

#### 3.1.1. Intra-rater Reliability (Intra- and Inter-session)

Intra-session and inter-session relative (ICCs and 95% confidence intervals (CIs)) and absolute (SEMs) reliability values for each condition are presented in Table 2. 

##### Relative Reliability

Intra-session intra-rater relative reliability (sessions 1 and 2) was excellent across all experts in the voluntary movement conditions in both healthy people and people with stroke (ICCs >0.90 and ICCs >0.84, respectively), with adequately restricted 95% CIs. For the imagined movement condition in healthy people, intra-session reliability within four experts was in the good range (ICCs 0.61–0.75) but had wide confidence intervals with the lower bound CIs extending into the poor range, and one expert was excellent (ICC = 0.90), with moderately-wide CIs. 

Inter-session intra-rater reliability (sessions 1 and 3) across experts was excellent in the voluntary movement conditions in both healthy people and people with stroke (ICCs >0.95 and ICCs >0.80, respectively), with narrower CIs for the voluntary movement condition in healthy people. ICCs for healthy imagined movement were good for four experts (ICCs 0.48–0.76), and poor for one expert (ICC = 0.05). For three out of the five experts, the lower bound CIs of the ICCs extended below 0 demonstrating very poor relative reliability.

##### Absolute Reliability

The intra-session intra-rater SEMs (sessions 1 and 2) for the voluntary movement conditions in both healthy people and people with stroke were fairly small across the five experts (22 ± 26 ms and 39 ± 24 ms, respectively), compared to the larger errors observed for the imagined movement condition in healthy people (119 ± 49 ms). A similar pattern across conditions was observed in the inter-session intra-rater SEMs (sessions 1 and 3), where SEMs for the voluntary movement conditions in both healthy people and people with stroke remained fairly small across experts (30 ± 15 ms and 59 ± 11 ms, respectively), but the imagined movement condition in healthy people produced a much larger measurement error (178 ± 66 ms).

#### 3.1.2. Inter-rater Reliability

Inter-rater relative (ICCs and 95% CIs) and absolute (SEMs) reliability values for each of the three conditions, appraised at each evaluation session, are presented in Table 3.

##### Relative Reliability

Inter-rater relative reliability within all three evaluation sessions was excellent for the voluntary movement condition in healthy people (ICCs > 0.95, with narrow 95% CIs). Similar results were observed in the voluntary movement condition in people with stroke (sessions 1 and 2) with the first two sessions in the excellent range (ICC > 0.84) and the third session almost excellent (ICC = 0.78), but with somewhat wider confidence intervals. Results for inter-rater reliability for the imagined movement condition in healthy people had much lower agreement, with ICC values ranging from poor to good across the three evaluation sessions with wide confidence intervals. Exemplar figures demonstrating substantial agreements and disagreements of PN labelling across the five experts for two different datasets can be found in the Supplementary Materials.

##### Absolute Reliability

Inter-rater SEMs across evaluation sessions for the voluntary movement conditions in both healthy people and people with stroke were akin to those reported for intra-rater inter-session reliability (40 ± 39 ms and 79 ± 67 ms, respectively), and the imagined movement condition in healthy people demonstrated very large SEMs (247 ± 180 ms).

### 3.2. Secondary Findings: Epoch Selection 

#### 3.2.1. Cosine Similarity across Conditions

The linear mixed-effects model revealed a significant difference in the cosine similarity index score across conditions (χ2 [2] = 28.67, *p* < 0.0021). Pairwise comparisons determined that cosine similarity index was significantly greater (t [27] = 4.32, *p* < 0.0005) in the voluntary movement in healthy people (0.39 ± 0.18) compared to the imagined movement in healthy people (0.27 ± 0.19) and significantly greater (t [27] = –4.89, *p* < 0.0001) in the voluntary movement in people with stroke (0.41 ± 0.18) compared to the imagined movement condition in healthy people (0.27 ± 0.19). There was no statistically significant difference in cosine similarity between voluntary movement conditions in people with stroke and healthy people (t [27] = –0.57, *p* < 0.0005).

#### 3.2.2. Intra-rater Reliability (Intra- and Inter-session)

**Figure 4:**
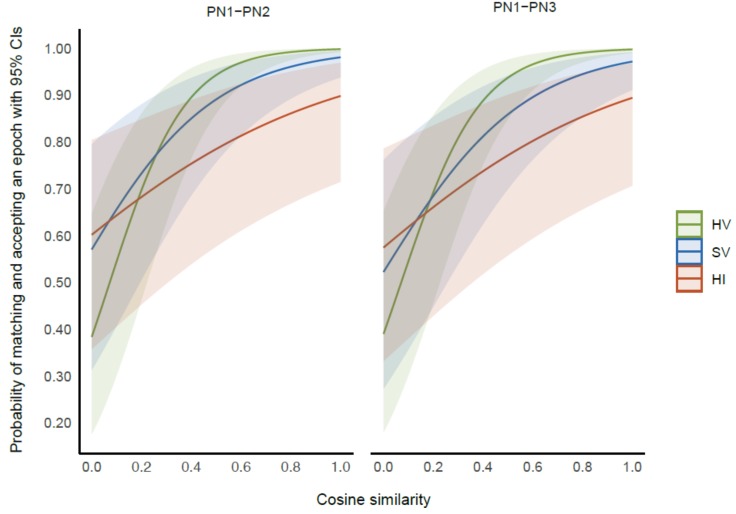
The relationship between the probability of experts obtaining a matched epoch and the cosine similarity. Data for each movement condition are presented (healthy voluntary (HV), stroke voluntary (SV), healthy imagined (HI)) with their 95% confidence intervals at intra-session (PN1 and PN2) and inter-session (PN1 and PN3) evaluations.

Results from the logistic mixed-effects model revealed a statistically significant interaction effect of cosine similarity and condition (χ2 [2] = 29.02, *p* < 0.0001) on the ability of experts to give matched responses. Linear log trends representing the association between cosine similarity (CS) and the log odds of epochs being matched were largest in the healthy voluntary movement condition (CS trend 6.41, 95% CI 5.17–7.65, *z =* 10.14, *p* < 0.0001). This indicates that for the voluntary movement condition in healthy people, for each unit increase in the cosine similarity index score, the log odds of getting a matched epoch increased by 6.41 times. This was followed by the voluntary movement condition in people with stroke (CS trend 3.58, 95% CI 2.38–4.67, *z =* 6.07, *p* < 0.0001) and imagined movement condition in healthy people (CS trend 1.80, 95% CI 0.73–2.86, *z =* 3.32, *p* < 0.0009). Figure 4 presents a graph demonstrating the relationship between the probability of experts obtaining a matched epoch and cosine similarity with 95% confidence intervals in each condition for intra-session (PN1 and PN2) and inter-session (PN1 and PN3) evaluations.

Results from the logistic mixed-effects model also revealed a statistically significant interaction effect of expert experience and condition (χ^2^ [2] = 60.5743, *p* < 0.0001) but within each condition the log trend was non-significant (voluntary movement condition in healthy people, trend 0.23, CI −0.09–0.5, *z =* 1.37, *p* = 0.1708; imagined movement condition in healthy people trend 0.15, CI −0.18–0.47, *z* = 0.86, *p* = 0.3884; voluntary movement condition in people with stroke, trend 0.13, CI −0.01–0.64 *z* = 1.89, *p* = 0.0580).

#### 3.2.3. Inter-Rater Reliability

**Figure 5:**
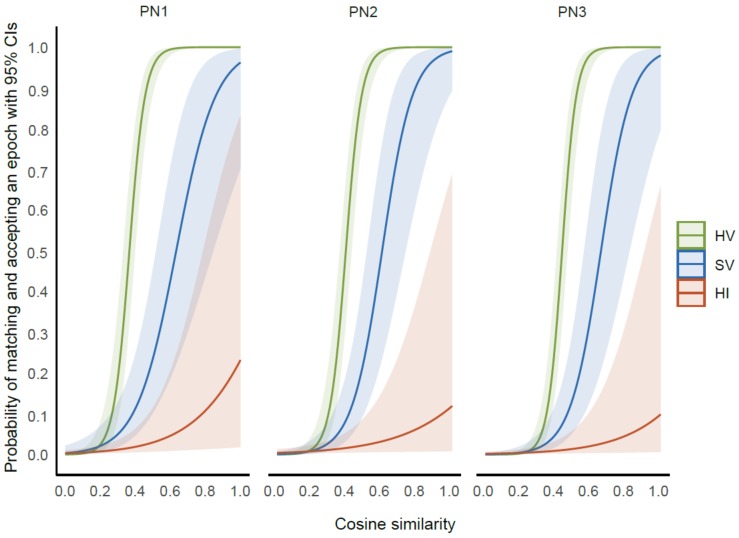
The relationship between the probability of experts obtaining a matched epoch and the cosine similarity data for each movement condition are presented (healthy voluntary (HV), stroke voluntary (SV), healthy imagined (HI)) with their 95% confidence intervals at each of the three evaluation sessions.

Results from the logistic mixed-effects model revealed a significant interaction effect of cosine similarity and condition (χ^2^ [2] = 30.74, *p* < 0.0001). There were no other statistically significant interaction effects (*p* < 0.05). Linear log trends representing the association between cosine similarity (CS) and the log odds of getting a matched epoch were largest in the voluntary movement condition in healthy people (CS trend 23.00, 95% CI 18.04–27.97, *z =* 9.08, *p* < 0.0001) indicating that for a unit increase in the CS index score the log odds of getting a matched epoch increased by 23 times. This was followed by the voluntary movement condition in people with stroke (CS trend 10.60, 95% CI 6.95–14.26, *z =* 5.69, *p* < 0.0001) and the imagined movement condition in healthy people (CS trend 4.04, 95% CI 1.14–6.94, *z = 2*.72, *p* = 0.0064). Figure 5 presents a graph demonstrating the relationship between the probability of all five experts obtaining a matched epoch and cosine similarity with 95% confidence intervals for each condition at each of the three evaluation sessions.

## 4. Discussion

This study is the first to our knowledge to assess the inter- and intra-rater (intra-session and inter-session) reliability of the manual labelling process used to determine the PN of the averaged MRCP. The main finding was that intra- and inter-rater reliability of the PN value were much higher in the voluntary movement conditions in both healthy people and people with stroke, than in the imagined movement condition in healthy people. ICC values for all voluntary movement conditions were excellent (ICC >0.8), except in the case of inter-rater reliability in the third session (PN3) in people with stroke, which approached excellent (ICC 0.78). When comparing the voluntary movement conditions in healthy people and people with stroke, the 95% confidence intervals for relative and absolute intra- and inter-rater reliability were consistently wider in the stroke group. This larger measurement error in the stroke data could be influenced by differences in the MRCP signals that are observed in people with stroke compared to healthy people, such as longer latencies and smaller amplitudes [44]. Differences in the MRCP signal may have also influenced the poorer reliability observed in the imagined movement condition in healthy people. While similar cortical regions are activated during imagined and voluntary movements [45,46,47,48], there is a lower level of excitation during imagined movements [49,50] as demonstrated by MRCPs with lower amplitudes and less defined peak negativities [44,51]. The possibility that differences in the morphology of the MRCP signals influences the reliability of the PN was explored in our secondary analysis.

The secondary analysis revealed a strong relationship between the cosine similarity and the probability of agreement in accepting epochs within and between experts. The cosine similarity reflects the similarity between the morphology of an MRCP epoch and the morphology of the average of all 50 epochs. The lower cosine similarity in imagined movement in healthy people, and to a lesser extent voluntary movement in people with stroke, suggests that the morphology of the MRCP changes from epoch to epoch to a greater extent in these conditions. Conditions with higher cosine similarity also had higher ICCs, for example, the voluntary movement condition in healthy people had the highest cosine similarity and the highest ICCs, whereas the imagined movement condition in healthy people had the lowest cosine similarity and the lowest ICCs. Collectively, these findings suggest that the morphology of the signal contributed to the consistency of epoch inclusion and may explain the differences in reliability for each movement condition observed in the primary analysis.

Our secondary intra-rater analysis also revealed a statistically significant interaction effect of expert experience with condition, on the ability of experts to give matched responses. However, within each condition this influence was found to be statistically non-significant. This may be because the study was not powered to detect an association between experience and ability to match epochs but suggests that further consideration should be given to experience, particularly when processing imagined movement data or data from people with neurological pathologies such as stroke. It is notable that in the imagined movement condition in healthy people, there appeared to be a within-session training effect. For example, the inter-rater reliability at the first session was poor (PN1 ICC = 0.40) but improved in the second session 30 min later (PN2 ICC = 0.76), and then decreased again in the third session one week later (PN3 ICC = 0.20). For intra-rater reliability of the imagined movement condition, ICCs were higher for within-session comparisons, which took place 30 min apart (ICC ranges 0.61–0.9), than the between-session comparisons, which took place one week apart (ICCs ranges 0.05–0.76). This possible training effect for evaluations that took place within 30 min of each other, could be due to several factors including: (1) the consistent order of presentation of epochs within each dataset at each session (only the conditions and datasets were randomized) and (2) experts had a longer exposure time to the datasets on day 1 with two evaluation sessions compared to one evaluation session a week later. Future work could assess whether evaluating an imagined movement dataset more than once improves reliability when manually labelling MRCP data.

The findings from this study suggest that the large measurement error observed in the manual labelling process of average MRCP PNs in the imagined movement condition in healthy people may contribute to the high between-participant variation observed in response to the ePAS intervention when delivered during imagined movements. If the timing of the pairings is a critical component to the success of the ePAS intervention, we propose that imagined movements may not be the most appropriate choice of movement condition. This illustrates the importance of considering reliability and measurement error in ERPs for both outcome measurements and the delivery of EEG-driven interventions.

### 4.1. Methodological Limitations

The generalizability of these results is subject to certain limitations. The EEG experts were from two academic centres and may not be representative of the EEG expert community working with ERPs. Expertise may influence the ability to match epochs and hence reliability, therefore the findings of this study may be less applicable to novice researchers who are processing ERP data. Intra- and inter-rater reliability for the voluntary movement condition in people with stroke was predominantly excellent (ICC > 0.8), but the smaller sample of people with chronic stroke may reduce the generalizability of these findings to the wider stroke population, as ICCs can be impacted by smaller samples where there is small between-participant variability [38,41]. Finally, this study examined the reliability of experts evaluating the same MRCP datasets on multiple occasions and not the test-rest reliability of different MRCP datasets collected from the same participants. While these study findings may be useful when considering the reliability of ERP data, they should be replicated in other lower or upper limb muscle groups, recorded on two or more occasions from the same participants or in other ERPs.

### 4.2. Future Recommendations

Future reliability studies should include experts from the wider ERP community and compare novice and expert evaluations. Future research should replicate the findings in a larger and more diverse sample of people with stroke and in other muscle groups, particularly if ePAS is seen as a potentially clinically viable neuromodulatory intervention for people with more acute stroke.

The results of this study may interest a wider community of researchers utilizing features of MRCPs during self-paced or cued paradigms to measure neurophysiological changes associated with motor performance and motor learning [52,53,54,55,56,57,58,59,60,61,62]. These results may also inform research into the development of automated labelling methods that can extract key features of ERPs. Rashid and colleagues [42] recently developed an automated method to identify MRCP features and highlighted the need for a reliable gold standard to which automated algorithms can be compared [63,64]. The present study is the first step towards providing reliable data for the comparison of manual labelling methods by EEG experts, with automated algorithms.

## 5. Conclusions

This study assessed the intra-rater and inter-rater reliability of EEG experts’ identification of the peak negativity (PN) feature from averaged movement related cortical potentials (MRCPs) obtained from healthy people and people with stroke. We found excellent inter- and intra-rater (intra-session and inter-session) reliability for the voluntary movement condition in both healthy people and people with stroke. In comparison, intra-rater and inter-rater reliability in the imagined movement condition in healthy people was low to moderate. Our secondary post hoc epoch analysis revealed that the morphology of MRCP signals influences the consistency of manual selection of epochs for inclusion within and between EEG experts. When the morphology of an MRCP epoch had greater similarity to the morphology of its average the probability of either a single expert or a group of experts selecting the same epochs for inclusion was much higher. Conditions with higher morphology similarity also had higher ICC values; for example, the voluntary movement condition in healthy people had the greatest morphology similarity and the highest ICCs, whereas the imagined movement condition in healthy people had the lowest morphology similarity and the lowest ICCs. The results from this study have implications for researchers evaluating MRCP data and potentially for those working with other ERP signals. This research may also provide a gold standard for the development of automated labelling methods of ERP features, as well as guidance for researchers who are using MRCPs as a neurophysiological measure of motor performance and motor learning in healthy and neurological populations.

## Figures and Tables

**Table 1:** Characteristics of study participants: healthy participants and participants with stroke.

	Healthy Participants	Participants with Stroke
Mean age (years)	28.6 (21–52)	67 (57–78)
Gender (males:females)	8:12	2:3
Lesion		
-Hemisphere (right:left)		3:2
-Type (ischemic:haemorrhagic)		4:1
Mean time since stroke (years)		7 (1–17)
Mean gait speed (m/s)		0.4 (0.2–0.75)
m/s = metres per second. Bracketed age and m/s data represent ranges.		

**Table 2:** Intra-rater reliability measures (intra-session and inter-session) of the labelled average MRCP PN for each of the three conditions.

Intra-session Reliability: PN1 and PN2						
	Healthy Voluntary DF		Healthy Imagined DF		Stroke Voluntary DF	
	**ICC**	**SEM**	**ICC**	**SEM**	**ICC**	**SEM**
E1	1.00 (0.99–1.00)	7.7	0.61 (0.34–0.89)	155.54	0.84 (0.46–0.96)	78.14
E2	0.99 (0.97–0.99)	18.9	0.90 (0.67–0.97)	63.55	0.91 (0.70–0.98)	46.98
E3	0.90 (0.67–0.97)	72.6	0.75 (0.30–0.93)	75.42	0.93 (0.75–0.98)	27.43
E4	1.00 (0.99–1.00)	6.99	0.63 (0.09–0.89)	120.38	1.00 (0.99–1.00)	3.84
E5	1.00 (0.99–1.00)	5.06	0.63 (0.06–0.89)	180.21	0.95 (0.80–0.99)	37.9
**Inter-session Reliability: PN1 and PN3**						
E1	0.99 (0.95–1.00)	19.61	0.55 (−0.03–0.86)	184.61	0.90 (0.65–0.98)	57.47
E2	0.98 (0.93–0.97)	30.56	0.76 (0.28–0.93)	115.10	0.94 (0.77–0.98)	41.22
E3	0.98 (0.93–1.00)	32.61	0.48 (−0.09–0.83)	116.01	0.85 (0.50–0.96)	55.81
E4	0.95 (0.83–0.99)	52.76	0.05 (−0.60–0.64)	279.2	0.80 (0.42–0.95)	72.81
E5	1.00 (0.98–1.00)	15.32	0.68 (0.10–0.91)	193.39	0.83 (0.46–0.96)	65.91

Intraclass correlation coefficients (ICCs) with lower and upper 95% confidence intervals and standard error of the measurements (SEMs) in milliseconds for each EEG expert. E = EEG expert number; DF = dorsiflexion; PN = peak negativity; PN1 = evaluation session 1; PN2 = evaluation session 2; PN3 = evaluation session 3.

**Table 3:** Inter-rater reliability measures of labelled average MRCP PNs for each of the three conditions.

Inter-rater reliability: PN1, PN2, PN3						
	Healthy Voluntary DF		Healthy Imagined DF		Stroke Voluntary DF	
	ICC	SEM	ICC	SEM	ICC	SEM
PN1	0.99 (0.97–1.00)	26.13	0.40 (0.14–0.74)	187.89	0.84 (0.67–0.95)	58.71
PN2	0.95 (0.88–0.98)	51.15	0.76 (0.53–0.92)	104.81	0.88 (0.74–0.96)	62.12
PN3	0.97 (0.92–0.99)	39.75	0.2 (0.00–0.60)	246.51	0.78 (0.67–0.87)	79.17

Intraclass correlation coefficients (ICCs) with lower and upper 95% confidence intervals and standard error of the measurements (SEMs) in milliseconds. PN = peak negativity; PN1 = evaluation session 1; PN2 = evaluation session 2; PN3 = evaluation session 3; DF = dorsiflexion.

## Supplementary Materials

The following are available online at https://www.mdpi.com/1424-8220/20/8/2427/s1, Figure S1: Averaged MRCPs from two example healthy participants performing imaginary movements. Five MRCP averages (different colors) were produced from the same dataset according to each expert’s included epochs. MRCP averages were offset for visual clarity (expert 1 = 0.5 uV, expert 2 = 1 uV, expert 3 = 1.5 uV, expert 4 = 2 uV, expert 5 = 2.5 uV). Sample number ‘1500’ corresponds to the onset of the cue to move. Each expert’s manually-labelled PN is circled. SD = standard deviation of samples across the five experts, Figure S2: Intra-rater analysis (intra-session and intersession). Possible options for an epoch *match* or *no match* by a single expert at two different time points. A = accepted; R = rejected, Figure S3: Inter-rater analysis. Possible options for an epoch *match* or *no match* across the five experts within a single evaluation session.
