# Supplementary material for: Intra- and Inter-Rater Reliability of Manual Feature Extraction Methods in Movement Related Cortical Potential Analysis
Source: Sensors (Basel). 2020 Apr 24;20(8):2427. doi: 10.3390/s20082427 (PMC7219488; doi:10.3390/s20082427)
Supplement: Supplementary file 1 [file sensors-20-02427-s001.pdf]

sensors

MDPI

Article

Intra- and Inter-rater Reliability of Manual Feature Extraction Methods in Movement Related Cortical Potential Analysis

Gemma Alder [1,*], Nada Signal [1], Usman Rashid [1], Sharon Olsen [1], Imran Khan Niazi [1,2] and Denise Taylor [1]

1 Health and Rehabilitation Research Institute, Auckland University of Technology, 0627, Auckland, New Zealand; nada.signal@aut.ac.nz (N.S.); usman.rashid@aut.ac.nz (U.R.); sharon.olsen@aut.ac.nz (S.O.); imran.niazi@nzchiro.co.nz(I.K.N.); denise.taylor@aut.ac.nz (D.T.);

2 Centre for Chiropractic Research, New Zealand College of Chiropractic, 1060, Auckland, New Zealand;

* Correspondence: gemma.alder@aut.ac.nz

Received: 2 March 2020; Accepted: 20 April 2020; Published: 24 April 2020

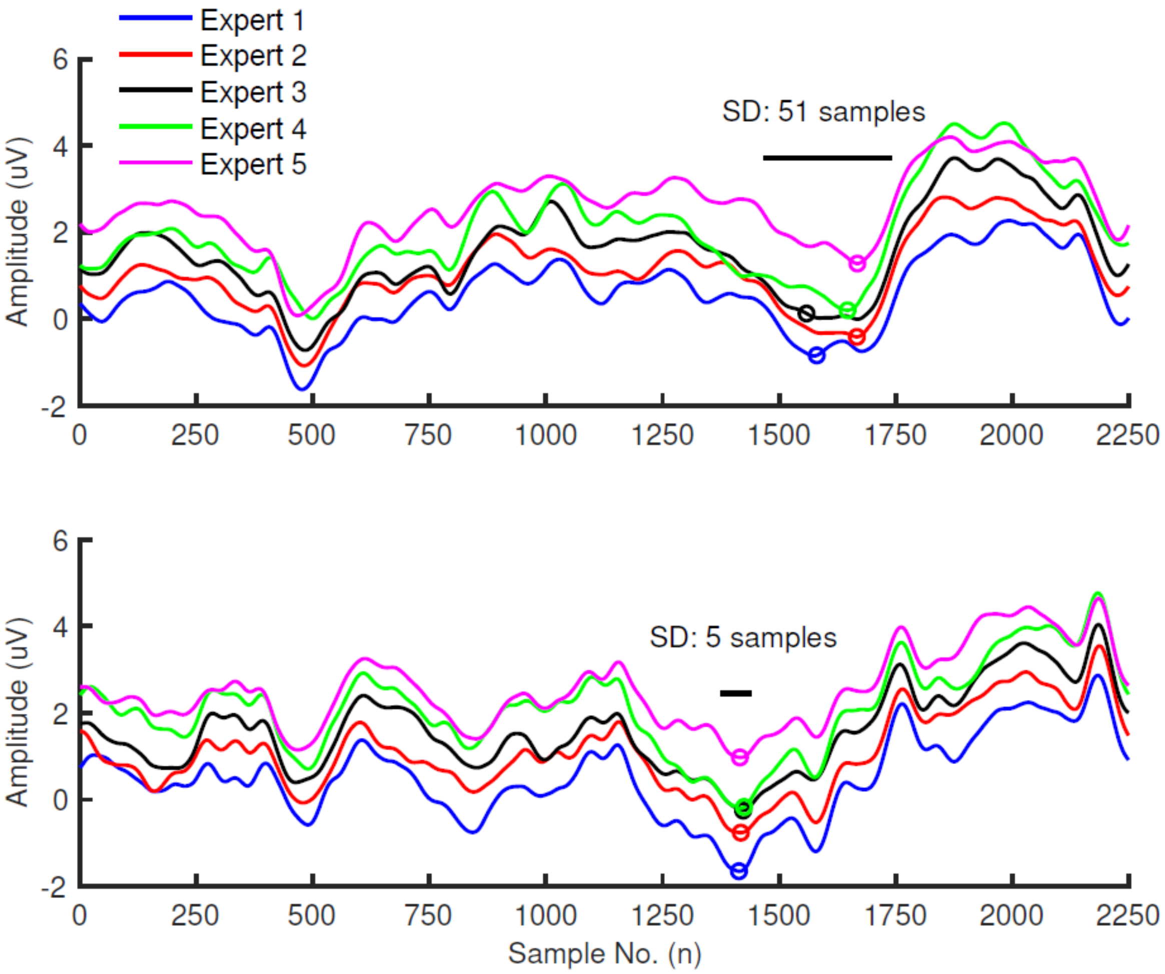

Figure S1. Averaged MRCPs from two example healthy participants performing imaginary movements. Five MRCP averages (different colors) were produced from the same dataset according to each expert's included epochs. MRCP averages were offset for visual clarity (expert 1 = 0.5 uV, expert 2 = 1 uV, expert 3 = 1.5 uV, expert 4 = 2 uV, expert 5 = 2.5 uV). Sample number '1500' corresponds to the onset of the cue to move. Each expert's manually-labelled PN is circled. SD = standard deviation of samples across the five experts.

The top graph in Figure S1 presents an imagined movement dataset where substantial disagreement between experts' average MRCP PN labelling was evident (SD = 51 samples). The bottom graph in Figure S1 presents a different imagined movement dataset where there was substantial agreement between experts' average MRCP PN labelling (SD = 5 samples).

Statistical Analysis Plan for Secondary Analysis: Epoch Selection

We investigated the influence of two factors: (1) morphology of the signal and (2) experience of an EEG expert on the ability of experts to provide the same response for inclusion of epochs. Epoch selection was defined as 'matched' if an expert chose to accept an epoch for inclusion at two different evaluation sessions (intra-rater: evaluation sessions 1 and 2 and 1 and 3) or if all five experts accepted the same epoch in a single evaluation session (inter-rater: evaluation sessions 1, 2 and 3).

Hypotheses:

We tested the following secondary null hypotheses:

(i) There is no relationship between the morphology of the epoch and the ability of an expert to accept the same epochs for inclusion across two evaluation sessions (intra-rater: evaluation sessions 1 and 2 and 1 and 3).
(ii) There is no relationship between the experience of an expert and their ability to accept the same epochs for inclusion across evaluation sessions (intra-rater: evaluation sessions 1 and 2 and 1 and 3).
(iii) There is no relationship between the morphology of the epoch and the ability of all experts to accept the same epochs for inclusion in a single evaluation session (inter-rater: evaluation sessions 1, 2 and 3).

Quantifying variables:

The morphology of the epochs was quantified using the cosine similarity index. This was defined as the similarity of a single epoch from a participant compared to the average MRCP of all 50 epochs of the same participant, which was considered a representation of the expected MRCP characteristics. The cosine similarity index within each condition was computed as follows [42]:

$$\text{Cosine similarity index} = \frac{\text{u.v}}{\|\text{u}\|\ \text{x}\|\text{v}\|}$$

where u and v are vectors, u is the average MRCP of all 50 epochs from a participant and v is a single epoch from the same participant. '.' represents the dot product between the two vectors and $\|.\|$ represents the L2 norm of a vector. The self-reported experience of experts working with MRCP signals was quantified in years.

We quantified '*matched*' and '*no match*' as binominal categorical variables. For the intra-rater analysis, we assigned *matched* when a single expert accepted the same epoch at both evaluation time points (i.e., evaluation sessions 1 and 2 (intra-session) or evaluation sessions 1 and 3 (inter-session)) and assigned *no match* when there was a mismatch in their epoch choice or both epochs were rejected. All possibilities for a given epoch are presented in Figure S2.

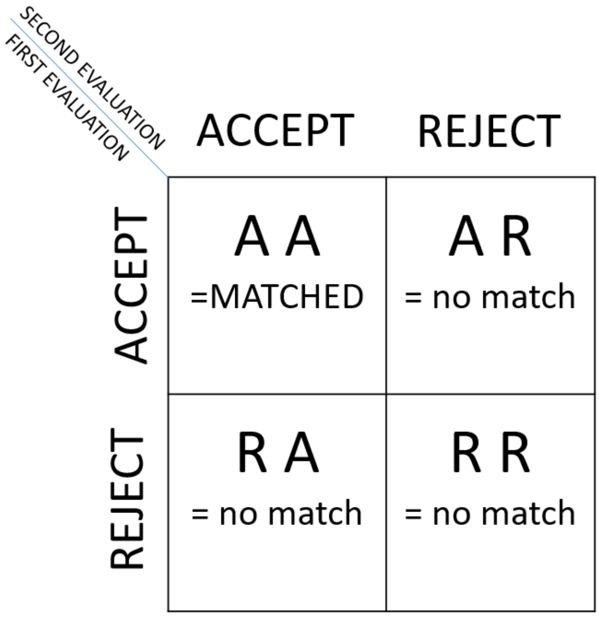

Figure S2. Intra-rater analysis (intra-session and intersession). Possible options for an epoch *match* or *no match* by a single expert at two different time points. A = accepted; R = rejected.

For the inter-rater analysis we assigned *matched* when all five experts accepted the same epoch at a single evaluation session (i.e., session 1, 2 or 3) and assigned *no match* in all other cases. For example, if four experts rejected an epoch and one expert accepted the epoch, we assigned a no match, or if all five experts rejected the epoch, we also assigned a no match. The possibilities for a given epoch are presented in Figure S3.

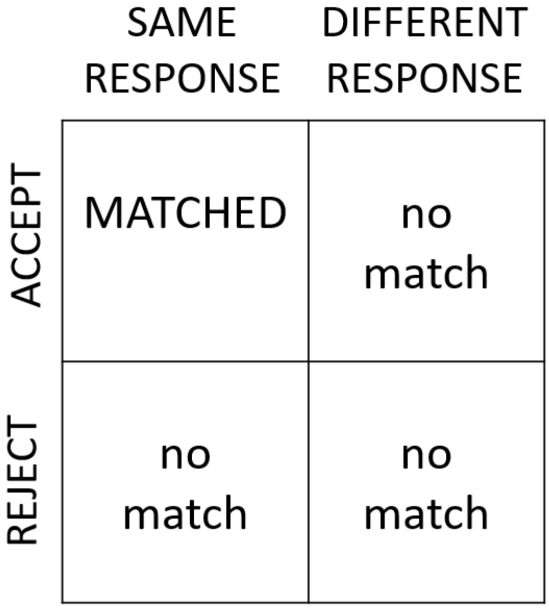

Figure S3. Inter-rater analysis. Possible options for an epoch *match* or *no match* across the five experts within a single evaluation session.

Intra-rater and inter-rater statistical models

Logistic mixed-effects models were set up in R version 3.5.1 (R Foundation for Statistical Computing, Vienna, Austria) and fitted using lme4 package version 1.1-17 [43]. For each condition

the significance level was set to $p < 0.05$. Car package version 3.0-0 [63] provided analysis of deviance tables for each of the models using the Anova function. Main effects and interactions were reported using Type III Wald Chi-squared tests. In the case of interactions, linear trends for cosine similarity and expert experience were obtained with emmeans package version 1.3.4 [64].

Intra-rater (intra- and inter-session) statistical model

Relates to hypotheses (i) and (ii)

To assess the influence of the morphology of epochs and expert experience on expert's ability to match epochs for inclusion at evaluation sessions 1 and 2 and 1 and 3 we used a logistic mixed-effects binominal regression model with a logit link.

Conditions, participants, single epochs, experts and time were entered as nominal categorical variables. Time had two levels: the first corresponding to epoch matches from evaluation session 1 and 2 and the second corresponding to epoch matches from evaluation sessions 1 and 3. Cosine similarity index and expert experience were entered as continuous variables. Cosine similarity index, time, expert experience and condition were treated as fixed effects. Participants, epochs nested under participants and experts were treated as random intercept effects.

$$\text{Matched\_status} \sim (\text{cosine\_similarity} \times \text{time} + \text{expert experience}) \times \text{condition} + (1| \text{participant/epoch}) + (1| \text{expert})$$

Inter-rater statistical model

Relates to hypothesis (iii)

To assess the influence of morphology on all five of the expert's ability to match epochs for inclusion at the same evaluation session we used a logistic mixed-effects binominal regression model with a logit link.

Condition, participants, single epochs and time were entered as nominal categorical variables. Time had three levels each corresponding to epoch matches from evaluation sessions 1, 2 and 3. Cosine similarity index was entered as a continuous variable. Condition and time were treated as fixed effects. Participants and epochs nested under participants were treated as random intercept effects.

$$\text{Matched\_status} \sim \text{cosine\_similarity} \times \text{time} \times \text{condition} + (1| \text{participant/epoch})$$

Cosine similarity statistical model

To assess cosine similarity across conditions a linear mixed-effects model with *Gaussian* family and an identify link was also set up in R version 3.5.1 (R Foundation for Statistical Computing, Vienna, Austria) and fitted using lme4 package version 1.1-17 [43]. For each condition the significance level was set to $p < 0.05$. In the case of significant findings for cosine similarity across conditions, pairwise *t*-tests using Tukey's method were performed and presented with cosine similarity means and standard deviations as mean ± SD for each condition.

Condition and epoch were entered as categorical variables and cosine similarity index as a continuous variable. Condition and epochs were treated as fixed effects. The cosine similarity of each study participant was modelled with a random intercept.

$$\text{cosine similarity} \sim \text{condition} \times \text{epoch} + (1|\ \text{participant})$$

References [63,64] are cited in the supplementary materials.

© 2020 by the authors. Licensee MDPI, Basel, Switzerland. This article is an open access article distributed under the terms and conditions of the Creative Commons Attribution (CC BY) license (http://creativecommons.org/licenses/by/4.0/).
